# Transforaminal endoscopic discectomy versus conventional microdiscectomy for lumbar discherniation: a systematic review and meta-analysis

**DOI:** 10.1186/s13018-018-0868-0

**Published:** 2018-07-05

**Authors:** Bin Zhang, Shen Liu, Jun Liu, Bingbing Yu, Wei Guo, Yongjin Li, Yang Liu, Wendong Ruan, Guangzhi Ning, Shiqing Feng

**Affiliations:** 1 Department of Orthopedics, Tianjin Medical University General Hospital, No. 154 Anshan Road, Heping District, Tianjin, 300052 People’s Republic of China; 2 Department of Orthopedics, First Affiliated Hospital of Gannan Medical University General Hospital, No. 23 Qingnian Road, Zhanggong District, Ganzhou, 341000 People’s Republic of China

**Keywords:** Transforaminal endoscopic discectomy, Conventional microdiscectomy, Meta-analysis

## Abstract

**Background:**

The open microdiscectomy is the most common surgical procedure for the decompression of radiculopathy caused by lumbar disk herniation. To date, a variety of minimally invasive (MI) techniques have been developed. In the last decades, endoscopic techniques have been developed to perform discectomy. The transforaminal endoscopic discectomy (TED) with posterolateral access evolved out of the development of endoscopic techniques.

**Methods:**

A systematic literature search was performed using the PubMed, EMBASE, and Cochrane Library databases for trials written in English. The randomized trials and observational studies that met our inclusion criteria were subsequently included. Two reviewers respectively extracted data and estimated the risk of bias. All statistical analyses were performed using Review Manager 5.3.

**Results:**

Five prospective and four retrospective studies involving 1527 patients were included. The results of the meta-analysis indicated that there were significant differences between the two groups in length of hospital stay (MD = − 8.41, 95% CI − 10.26, − 6.56; *p* value < 0.00001). However, there were no significant differences in the leg visual analog scale (VAS) scores, the Oswestry Disability Index (ODI) scores, and the incidence of complications and recurrence.

**Conclusions:**

The transforaminal endoscopic discectomy is superior to open microdiscectomy in the length of hospital stay. However, there were no differences in leg pain, functional recovery, and incidence of complications between TED and MD in treating LDH.

## Background

Lumbar disk herniation (LDH) is a common medical condition with a pathological process that leads to spinal surgery. The fibrous ring of an intervertebral disk is fractured and allows the soft central portion, the nucleus pulposus, to bulge out beyond the damaged fibrous rings. LDH is considered to be the most prevalent spinal disk herniation and always causes a series of signs and symptoms. One of the most challenging medical problems is sciatica symptoms. Sciatica affects millions of individuals worldwide [1]. The nerve root compression caused by the bulge of the nucleus pulposus and the secondary inflammatory reaction represent two crucial factors that result in lumbosacral radicular syndrome [2]. With the aggravation of LDH, incontinence may develop [3].

Currently, early conservative treatment is used when the symptoms are not serious. However, surgery is adopted when conservative treatment fails, or complaints worsen over time [4, 5]. In 1934, lumbar disk herniation was the first condition treated surgically by performing an open laminectomy and discectomy [6]. With the introduction of the microscope, the open lumbar discectomy was refined into open microdiscectomy (MD) [7]. Currently, the open microdiscectomy is the most common surgical procedure for decompression of radiculopathy caused by lumbar disk herniation [8]. Since then, a variety of minimally invasive (MI) techniques have been developed. The minimally invasive techniques provide a similar view with a small incision and better cosmetic results [9, 10]. In the last decades, endoscopic techniques have been developed to perform discectomy under direct view and local anesthesia. The transforaminal endoscopic discectomy (TED) with posterolateral access evolved out of the development of endoscopic techniques [11–15]. The lateral access of transforaminal endoscopic discectomy to the spinal canal under continuous visualization has been developed since the late 1990s [16].

The indications for transforaminal endoscopic treatment are similar to classical open microdiscectomy procedures [17, 18]. However, a controversy remains over whether TED or MD should be utilized in clinical practice. It is therefore necessary to compare the clinical efficacies of different procedures to generate data that can be used to formulate clinical guidelines. Our goal was to systematically review, grade, and perform a meta-analysis of existing comparative studies. In this review, we compared the safety and efficacy of TED and MD for treating LDH patients.

## Methods

### Study design

The standards set by the Preferred Reporting Items for Systematic Reviews and Meta-Analyses (PRISMA) guidelines were used to construct this systematic review. The 27-item checklist and 4-phase flow diagram of PRISMA were both consulted.

### Literature search

The PubMed, EMBASE, and Cochrane Library databases were searched up to January 2017 to identify studies comparing transforaminal endoscopic discectomy with microdiscectomy for the treatment of lumbar disk herniation. The search terms included “transforaminal endoscopic discectomy,” “microdiscectomy,” “endoscopic,” “minimally invasive,” and “lumbar disk herniation.”

References from each article directly comparing the two kinds of surgeries, in addition to review articles discussing the safety and efficacy of the two procedures, were cross-referenced to identify additional relevant studies.

### Inclusion and exclusion criteria

For inclusion in the systematic review, the articles were required to meet the following eligibility criteria: (1) patients suffering from lumbar disk herniation; (2) papers reporting the results of clinical studies evaluating transforaminal endoscopic discectomy and microdiscectomy; (3) patients followed for a minimum of 2 weeks; and (4) papers published in English prior to January 2017. Randomized controlled trials (RCTs) were identified as the primary studies for analysis. For inclusion in statistical analysis, the patients in a particular study must have been randomized to either TED or MD groups. Studies were excluded from the analysis if they included patients who had an infection, traumatic fracture, previous spinal surgery at the same disk level, and spinal stenosis among other conditions. The inclusion criteria for each study are listed in Table 1.

**Table 1:** Summary of study criteria—prospective studies and retrospective studies

Study	Study type	Sample size	Av. age	Mean duration of follow-up (months)	Gender (M/F)	Level
Hermantin et al. [20]	RCT	60	39 vs. 40	31(19–42) vs. 32(21–42)	22:8/17:13	L2-L3L3-L4L4-L5L5-S1
Mayer and Brock [21]	RCT	40	39.8 ± 10.4 vs. 42.7 ± 10	6.9	12:8/14:6	L2-L3L3-L4L4-L5
Ruetten et al. [10]	RCT	129	43	24	–	L1-L2L2-L3L3-L4L4-L5L5-S1
Gibson et al. [22]	RCT	140	42.0 ± 9 vs. 39 ± 9	24	30:40/40:30	L3-L4L4-L5L5-S1
Akçakaya et al. [27]	RCT	30	44.1	–	–	–
Kim et al. [23]	Retro	902	34.9 vs. 44.4	23.6	188:107/392:215	L1-L2L2-L3L3-L4L4-L5L5-S1
Lee et al. [24]	Retro	60	39.3 vs. 39.6	38.2(32–45) vs. 36.8(35–42)	22: 8/22: 8	L4-L5L5-S1
Ahn et al. [25]	Retro	66	22.41 ± 1.68 vs. 22.18 ± 1.51	13.69 + 1.26 vs. 13.41 + 1.02	32:0/34:0	L4-L5
Hsu et al. [26]	Retro	100	–	20.4	–	L1-L2L2-L3L3-L4L4-L5

### Risk of bias

The risk of bias of the Cochrane Handbook for Systematic Reviews of Interventions was evaluated by using the risk of bias tool implemented in Review Manager 5.3. The included RCTs were evaluated for the risk of bias, which included assessments of adequate sequence generation, allocation of concealment, blinding, incomplete outcome data, and freedom from other biases. The judgment of each entry involved assessing the risk of bias as “low risk,” “high risk,” or “unclear risk,” indicating either a lack of information or uncertainty over the potential for bias. Two reviewers independently assessed each RCT, and any disagreements were resolved by discussion and consensus.

### Data extraction

Two authors independently extracted the following data. Any disagreements were resolved via discussion among the three reviewers. The data extracted from the studies included the following: study characteristics, types of interventions, follow-up duration, and outcome parameters.

### Outcome measures

The “degree of pain relief” (visual or verbal analog pain scale (VAS) score) and the functional improvement (Oswestry Disability Index (ODI)) were the primary outcome measures of the effectiveness of the surgeries. The secondary outcome measures were average duration of surgery, complications, hospital stay, recurrence, and satisfactory outcome.

### Statistical analysis

The data were collected and analyzed using Review Manager 5.3. Differences in pain, functional improvement, average duration of surgery, and hospital stay between the TED and MD groups were analyzed using the independent samples *t* test under a random-effects model. Risk ratios (RRs) and 95% confidence intervals (CIs) were used to evaluate the dichotomous outcomes, such as the incidence of complications. The differences are displayed using a forest plot. The *I*^2^ statistic [19] (ranging from 0 to 100%) was applied to quantify between-study heterogeneity that was not attributed to chance (*I*^2^ = 0–25%, no heterogeneity; *I*^2^ = 25–50%, moderate heterogeneity; *I*^2^ = 50–75%, large heterogeneity; and *I*^2^ = 75–100%, extreme heterogeneity). A *p* value < 0.05 was considered statistically significant.

## Results

### Literature search

**Fig. 1:**
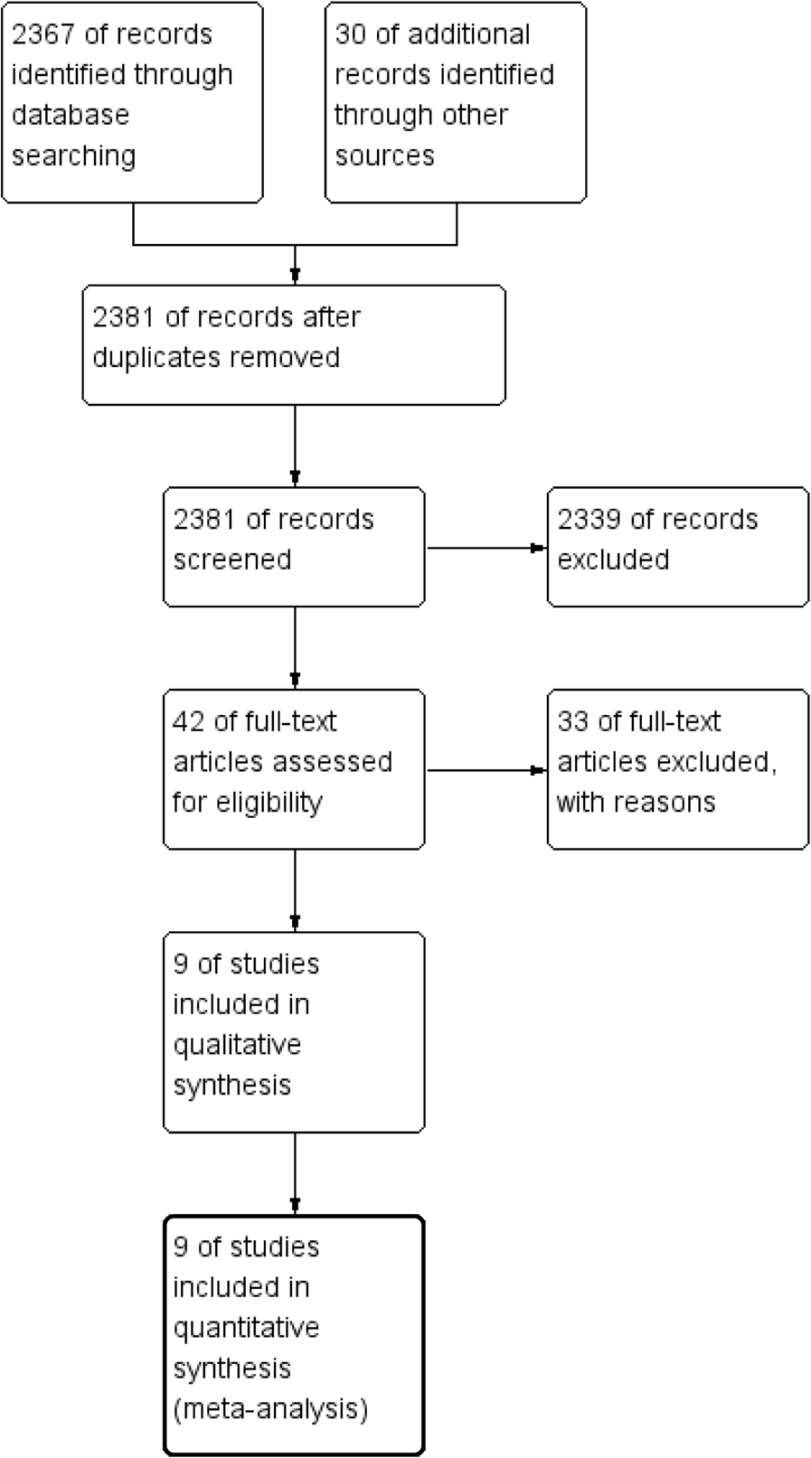
Flow diagram of the search and selection criteria for inclusion in this meta-analysis

A total of 2397 records were identified through the PubMed, EMBASE, and the Cochrane Library database. Following the exclusion of 369 duplicate items, 2381 articles were screened for review, and 42 that met the inclusion criteria were selected. A total of 33 full-text articles were excluded due to either the absence of a comparison between transforaminal endoscopic discectomy and conventional microdiscectomy or the absence of an appropriate statistical analysis. Nine studies [10, 20–27] were ultimately included in the meta-analysis (Fig. 1).

### Risk of bias in included studies

**Fig. 2:**
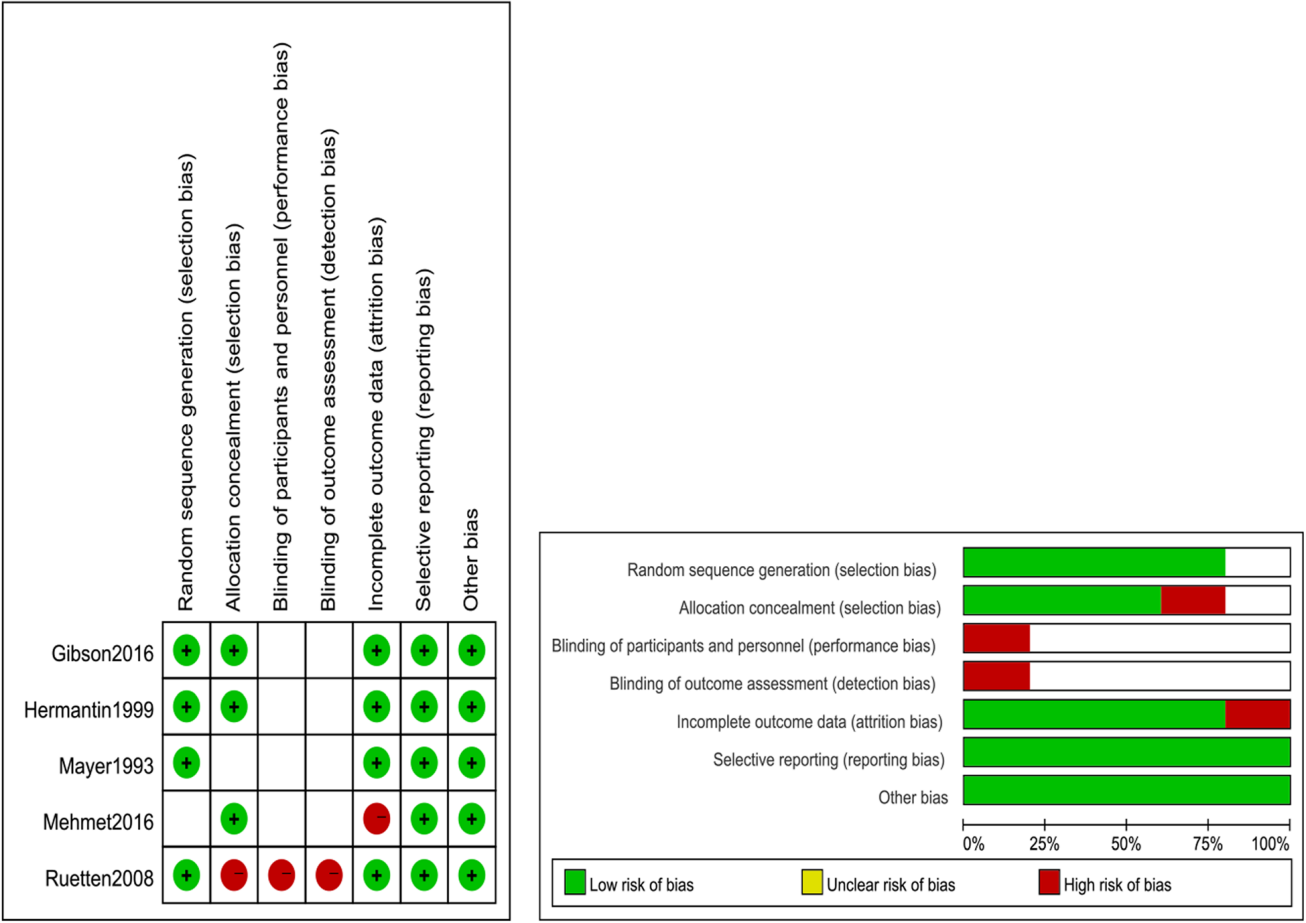
Risk of bias assessment of each included study. **a** Risk of bias graph. **b** Risk of bias summary

We used the risk of bias tool implemented in Review Manager 5.3 to evaluate the risk of bias of the Cochrane Handbook for Systematic Reviews of Interventions. The particular information of the risk of bias of the included articles is demonstrated in Fig. 2. Four [10, 20–22] of five studies comprehensively described the generation of a randomized sequence. The patients were not blinded to the treatment allocation in one study [10], which consisted of four indistinct studies [20–22, 27]. One article [27] displayed a high risk of bias for the incomplete outcomes. The rest of the included articles displayed a low risk of bias for the incomplete outcomes, selective outcome reporting, and other biases.

### Demographics of the studies included in the review

Five [10, 20–22, 27] of the nine studies were prospective studies, and four [24–26, 28, 29] were retrospective studies. These studies included 1527 patients, 399 of whom were included in the prospective studies, and 1128 of whom were included in the retrospective studies. Statistical analysis was performed in the five prospective studies and four retrospective studies. Differences in age, gender, and level were noted; however, these differences were not statistically significant. Follow-up periods ranged from 6.9 to 24 months in duration (Table 1).

### Outcome analyses: leg pain

**Fig. 3:**
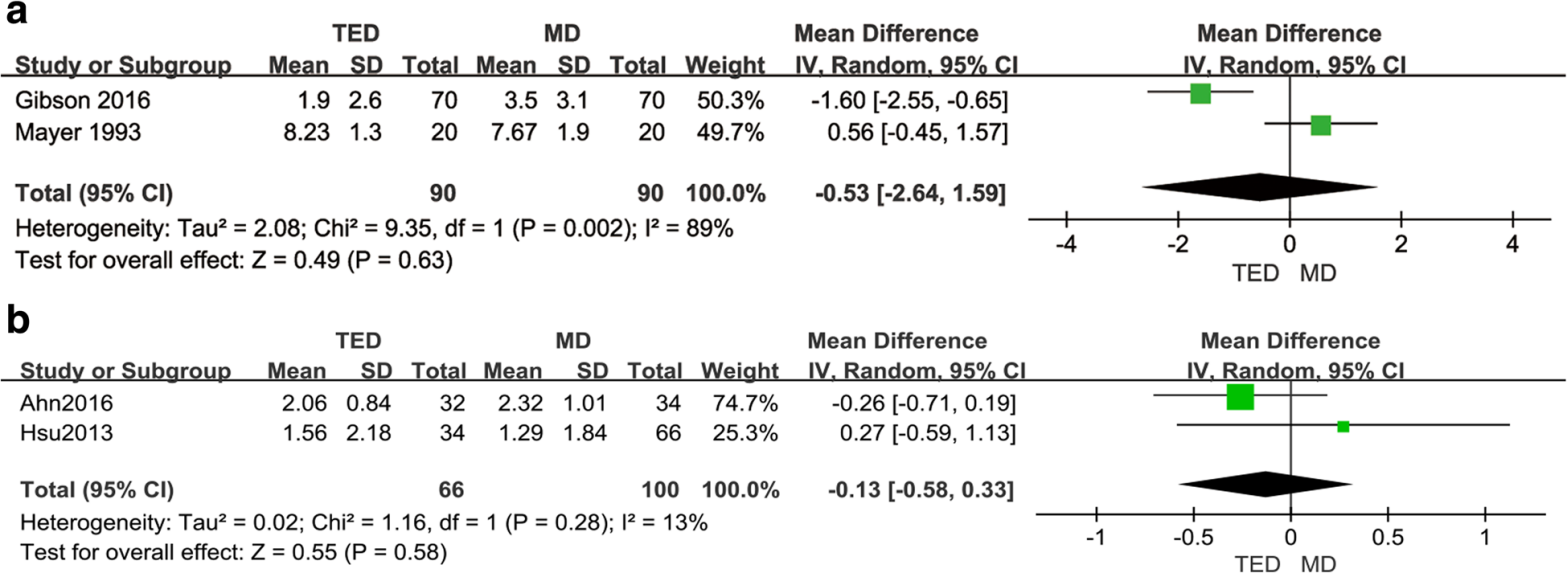
A comparison of MD vs. TED with respect to pain level following surgery in RCTs (**a**) and retrospective studies (**b**)

VAS scores were available in two of the RCT studies. Mayer and Brock [21] show that the VAS scores were 8.23 ± 1.3 and 7.67 ± 1.9 in the TED and MD groups at 2 years postoperation, respectively. Both groups showed a significant difference between preoperative and postoperative scores. Gibson et al. [22] showed that the VAS scores were 1.9 ± 2.6 and 3.5 ± 3.1 in the TED and MD groups at 2 years postoperation, respectively. Meta-analyses were performed in these two studies. Although the heterogeneity was high (*I*^2^ up to 89%), slightly better leg pain relief was observed in the TED group at 2 years and no differences were noted after 2 years of follow-up (Fig. 3a).

Two retrospective studies [25, 26] reported VAS scores (Fig. 3b). Meta-analyses were also performed in these two studies. Although the *I*^2^ was low, the credibility was not high. Similar outcomes were reported in these two retrospective studies compared to the RCT studies. No significant differences were observed between the TED and MD groups (SMD = − 0.13, 95% CI − 0.58, 0.33; *p* = 0.58).

### Outcome analyses: functional recovery

**Fig. 4:**
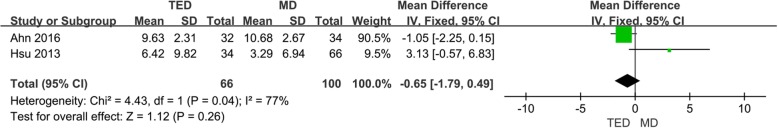
A comparison of MD vs. TED with respect to functional recovery following surgery in retrospective studies

Function was measured using an ODI. Only two retrospective studies [25, 26, 28] had reported the ODI scores. Ahn et al. [25] reported that the ODI scores were 9.63 ± 2.31 and 10.68 ± 2.67 in the TED and MD groups, respectively. Hsu et al. [26] reported that the ODI was 6.42 ± 9.82 and 3.29 ± 6.94 in the TED and MD groups, respectively. There were no significant differences between the TED and MD groups (Fig. 4).

### Operative time

**Fig. 5:**
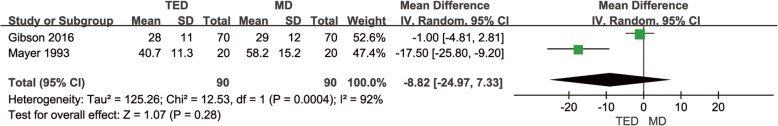
A comparison of MD vs. TED with respect to operative time following surgery in RCT studies

The average duration of surgery was available in two of the RCT studies. Mayer and Brock [21] showed that the operative time was 40.7 ± 11.3 and 58.2 ± 15.2 min in the TED and MD groups, respectively. Gibson et al. [22] showed that the VAS scores were 28 ± 11 and 29 ± 12 min in the TED and MD groups, respectively. Meta-analyses were performed in these two studies. Although a shorter operative time was observed in the TED group, there were no significant differences between the two groups (Fig. 5).

### Stay in hospital

**Fig. 6:**
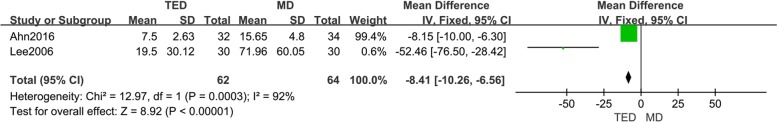
A comparison of MD vs. TED with respect to length of hospital stay following surgery in retrospective studies

Only two retrospective studies [24, 25] reported hospital stay (Fig. 6). Meta-analyses were performed in these two studies. Both studies reported a shorter hospital stay in the TED group vs. the MD group. The time was 19.5 ± 30.12 vs. 71.96 ± 60.05 and 7.5 ± 2.63 vs. 15.65 ± 4.8 h, respectively. The differences between the TED and MD groups was statistically significant (MD = − 8.41, 95% CI − 10.26, − 6.56; *p* < 0.00001).

### Complications

**Fig. 7:**
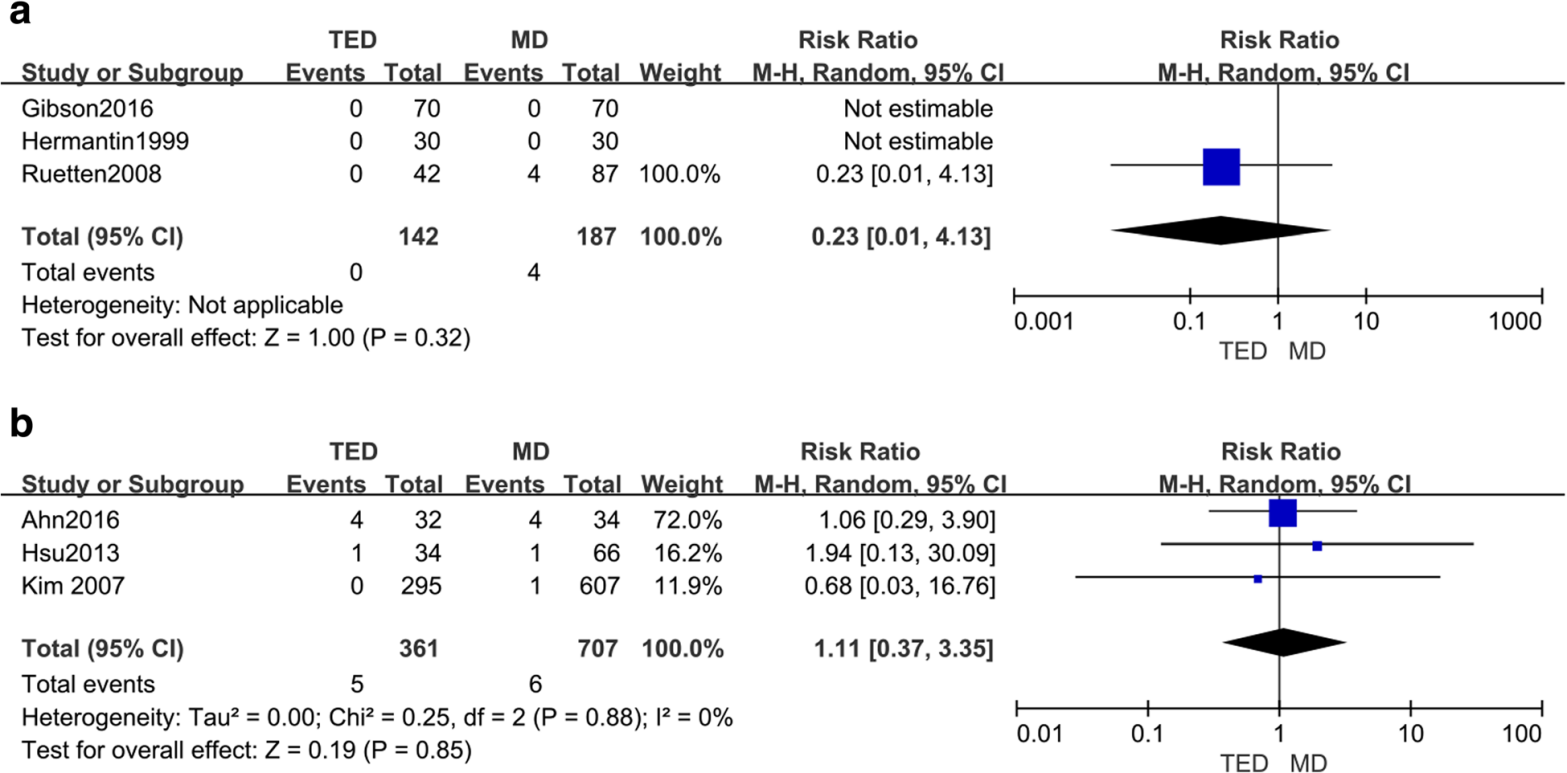
A comparison of MD vs. TED with respect to complications following surgery in RCTs (**a**) and retrospective studies (**b**)

Both the RCT and retrospective studies recorded the postoperative complications (Fig. 7a). The conditions related to the complications were available in three of the RCT studies [10, 20, 22]. There were no complications reported in the articles of Hermantin et al. [20] and Gibson et al. [22]. No complications were reported in the TED group, but four complications and three complications were reported in the MD group [10, 22]. No significant differences were reported between the TED and MD groups (RR = 0.23, 95% CI 0.01, 4.13; *p* = 0.32).

Three retrospective studies [23, 25, 26] reported complications (Fig. 7b). Meta-analyses were also performed in these three studies. Similar outcomes were reported in these three retrospective studies compared to the RCT studies. However, the rate of complications was slightly higher in the MD group; differences between the TED and MD groups were not statistically significant.

### Rate of recurrence

**Fig. 8:**
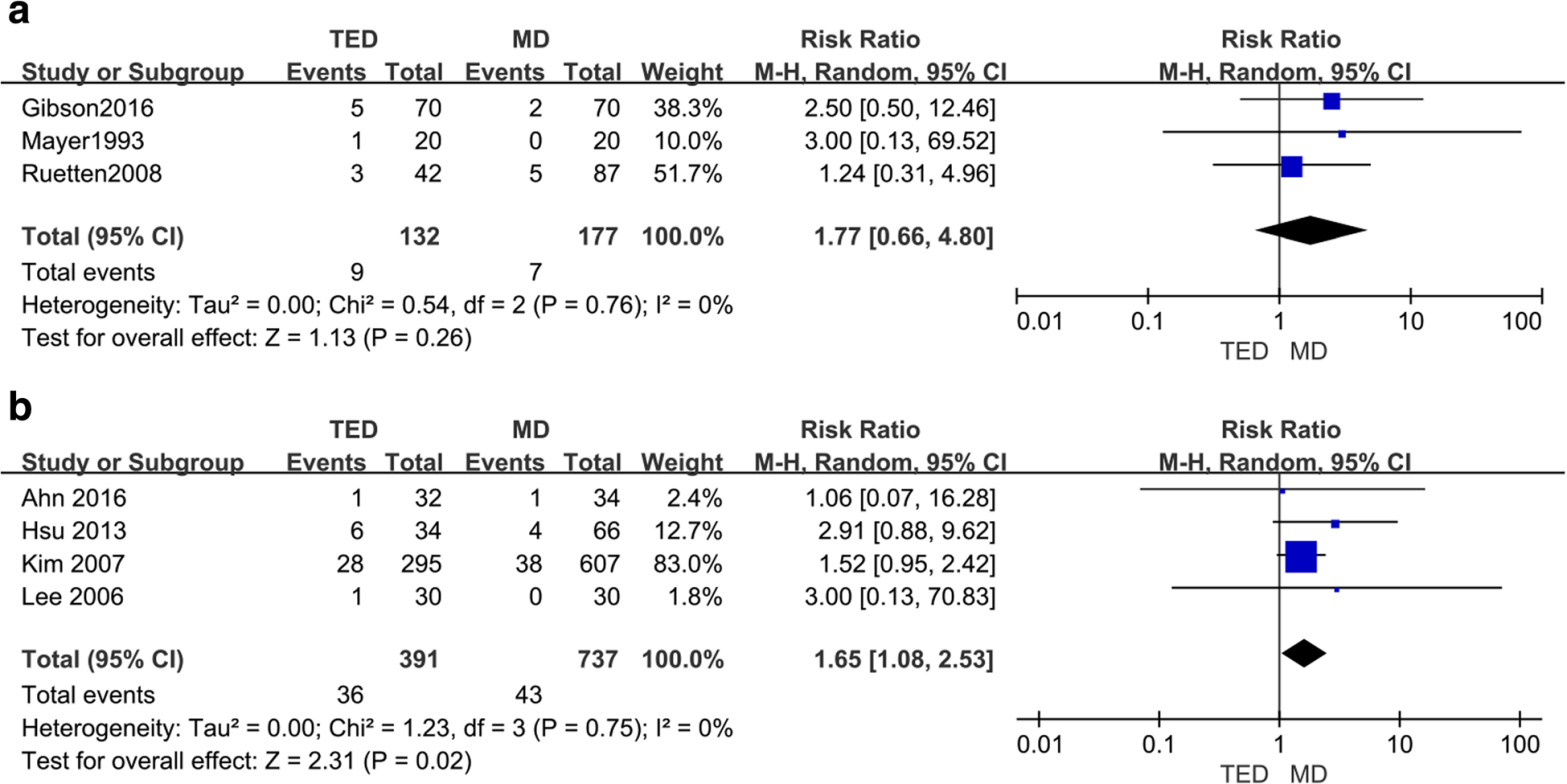
A comparison of MD vs. TED with respect to the rate of recurrence following surgery in RCTs (**a**) and retrospective studies (**b**)

The recurrence was recorded in three of the RCT studies [10, 21, 22]. All the RCT studies reported a higher rate of recurrence in the TED group. No significant differences were observed between the TED and MD groups (RR = 1.77, 95% CI 0.66, 4.8; *p* = 0.26) (Fig. 8a).

Four retrospective studies [23–26] reported recurrence. Similar outcomes were observed in these four retrospective studies compared to the RCT studies. A higher rate of recurrence was observed in the TED group, and the difference between the TED and MD groups was statistically significant (RR = 1.65, 95% CI 1.08, 2.53; *p* = 0.02) (Fig. 8b).

## Discussion

Lumbar open microdiscectomy is a popular procedure for the surgical treatment of lumbar disk herniation [28]. However, the open microdiscectomy surgery often requires a large incision to provide optimal vision. During the surgery, the paravertebral muscles are retracted, and the spinal lamina and facet joint are removed. This surgery can cause scarring and instability of the spine, which causes clinical symptoms in 10% or more of patients [29]. The transforaminal endoscopic discectomy was developed in the 1990s. Compared to the open microdiscectomy, it has several advantages. The TED can be performed under local anesthesia; thus, the rate of anesthesia-associated complications is low. The risk of scar formation and instability of the spine is also decreased [30–33]. A review [34] of the comparisons between TED and MD showed that TED was strongly favored. It is therefore necessary to compare the clinical efficacies of the different procedures to generate data that help surgeons make clinical decisions and develop optimal treatments.

We summarized the results of studies comparing transforaminal endoscopic discectomy and open microdiscectomy and performed a meta-analysis to compare the effectiveness and safety of these two surgeries for treating lumbar disk herniation. We analyzed the effectiveness of these two procedures by evaluating improvements in patients’ pain, functional scores, average duration of surgery, and hospital stay. We also analyzed the safety of these two procedures by evaluating complications and recurrence of LDH. We included five prospective studies and four retrospective studies involving 1527 patients in our analysis (Table 2).

**Table 2:** Summary of information of the prospective studies and retrospective studies

Study	Low-back pain	Leg pain	ODI	Recurrence	Hospital stay	Complications	Average duration of surgery (min)
Hermantin et al. [20]	–	1.2 vs. 1.9	–	–	–	0 vs. 0	–
Mayer and Brock [21]	10/19 vs. 15/20	8.23 ± 1.3 vs. 7.67 ± 1.9	–	1/20 vs. 0/20	–	–	40.7 ± 11.3 vs. 58.2 ± 15.2
Ruetten et al. [10]	–	–	–	3 vs. 5	–	0 vs. 4	–
Gibson et al. [22]	2.50 ± 2.5 vs. 3.0 ± 2.8	1.9 ± 2.6 vs. 3.5 ± 3.1	18 ± 17 vs. 22 ± 20	5 vs. 2	0.7 ± 0.7 vs. 1.4 ± 1.3 days	0 vs. 0	28 ± 11 vs. 29 ± 12
Mehmet [27]	–	–	–	–	1.13 vs. 1.2 days	–	94 vs. 71
Kim et al. [23]	–	–	–	28/295 vs. 38/607	–	0 vs. 1	–
Lee et al. [24]	–	–	–	1 vs. 0	19.5 ± 30.12 vs. 71.9 ± 60.05 h	–	42.6 ± 14.21 vs. 65.1 ± 23.17
Ahn et al. [25]	2.50 ± 0.62 vs. 2.91 ± 0.67	2.06 ± 0.84 vs. 2.32 ± 1.01	9.63 ± 2.31 vs. 10.68 ± 2.67	1 vs. 1	7.50 ± 2.63 vs. 15.65 ± 4.80 h	4 vs. 4	48.66 ± 6.45 vs. 53.71 ± 8.49
Hsu et al. [26]		1.56 ± 2.18 vs. 1.29 ± 1.84	6.42 ± 9.82 vs. 3.29 ± 6.94	6 vs. 4		1 vs. 1	86.5 ± 45.9 vs. 48.1 ± 9.2

No significant difference in both leg pain and function recovery was observed between TED and MD. Both RCTs and retrospective studies support the evidence that the transmuscular approach to the transforaminal endoscopic discectomy is as effective as the conventional open microdiscectomy requiring paravertebral muscle retraction. It can be explained that the clinical symptoms were caused by the decompression of the nerve root due to the herniated disk [35]. Both surgical procedures can remove nerve compression. However, several studies [36] suggested that clinical outcomes were associated with paravertebral muscle injury. Additionally, some factors such as different sampling dates, different peri- and intraoperative procedures, and different surgeons may have influenced the clinical outcomes.

The results in operative times and length of hospital stay were difficult to interpret. Although the operative times of the TED group was slightly shorter than the MD group, no significant difference was observed between TED and MD groups. The differences in how operative time was defined are important. Whether the anesthesia time was considered into the operative time, it had a large influence on operative time. Moreover, the variability in the techniques used was also a factor [37]. The length of hospital stay was much shorter in the TED group compared to that in the MD group. Transforaminal endoscopic discectomy may be associated with less muscle damage, among other outcomes [38], which allows the early recovery of patients. Additionally, economic factors should be considered.

In this meta-analysis, no significant difference in rates of total complications was observed between the two groups. Some studies [25, 26] suggested that the TED approach would be associated with a higher rate of complications. A limited surgical exposure leads to difficulty in surgery, and therefore, it is easier to cause nerve damage and other complications [39]. However, other research suggests the opposite because the small incision and minimal internal tissue damage make it possible for a shorter recovery period and minimization of scar tissue [40, 41]. No significant difference in rates of recurrence was observed between the two groups.

One limitation of this study was the small number of RCTs. Although five RCTs were included in this article, different outcomes could only be extracted from a few studies. It made assessing the effectiveness and safety of the interventions on the different surgical approaches difficult. Another limitation of this review was that clinical heterogeneity, which cannot be resolved completely, may be associated with inconsistency of outcomes.

## Conclusions

Our study demonstrated that transforaminal endoscopic discectomy was superior to open microdiscectomy in the length of hospital stay. However, there was no difference in leg pain, functional recovery, and incidence of complications between TED and MD in treating LDH. Prior to selecting a surgical procedure for the management of LDH, the benefits and risks of the procedure discussed herein must be taken into consideration. Additional studies must be performed to guide the clinical decision-making process.

## Abbreviations

LDH: Lumbar disk herniation
MD: Microdiscectomy
MI: Minimally invasive
PRISMA: Preferred Reporting Items for Systematic Reviews and Meta-Analyses
SMD: Standardized mean differences
TED: Transforaminal endoscopic discectomy
